# Editorial: Multimodality imaging techniques in PD and atypical Parkinsonism

**DOI:** 10.3389/fneur.2024.1370437

**Published:** 2024-02-13

**Authors:** Tianbin Song, Binbin Nie

**Affiliations:** ^1^Department of Radiology and Nuclear Medicine, Xuanwu Hospital, Capital Medical University, Beijing, China; ^2^Beijing Key Laboratory of Magnetic Resonance Imaging and Brain Informatics, Beijing, China; ^3^Beijing Engineering Research Center of Radiographic Techniques and Equipment, Institute of High Energy Physics, Chinese Academy of Sciences, Beijing, China

**Keywords:** PD, Parkinsonism, PET, MRI, neuromelanin, DAT-PET, ^18^F-FDG

In this editorial, we provide a summary of the articles that have contributed to the Research Topic “*Multimodality imaging techniques in PD and atypical Parkinsonism*” in the journal Frontiers in Neurology.

Parkinson's disease (PD) is the second most common neurodegenerative disorder, known for its variety of motor and non-motor symptoms (1). Molecular imaging technologies like PET/CT and PET/MR have seen significant advancements in recent years, making them the most valuable tools for diagnosing PD today and in the near future. In this Research Topic on “*Multimodality imaging techniques in PD and atypical Parkinsonism*,” researchers have provided distinct perspectives and innovative solutions for this challenge.

Diagnosing prodromal PD in clinical settings poses significant challenges. Over the past decade, several studies have concentrated on the prodromal stage of PD and have identified potential biomarkers for its diagnosis. Jackson et al. utilized the PD progression markers initiative (PPMI) to compare the prevalence of clinical markers of PD with prodromal PD. Their findings indicated that symptoms such as constipation and speech difficulty could potentially serve as predictors of prodromal PD and PD, respectively. This suggests that clinical markers could be valuable tools in identifying individuals at higher risk of developing PD.

Neuromelanin-sensitive MRI is capable of accurately measuring nigral damage and distinguishing PD from healthy subjects. In the substantia nigra, iron deposition is typically concentrated in the reticular zone, while neuromelanin is predominantly concentrated in the dense zone. In PD patients, the content of neuromelanin in the dense zone of the substantia nigra decreases and iron deposition increases, reflecting the degeneration process of dopamine neurons (2). Cao et al. discovered that the amplitude, phase value, and R2^*^ value of upper, middle, and low segments of right substantia nigra compact zones in PD patients differed significantly from those in the control groups. The R2^*^ value of the substantia nigra dense zone was correlated with the H-Y grade, indicating that quantitative iron deposition in the substantia nigra dense zone could be a sensitive imaging biomarker for early diagnosis, assessment of severity, and follow-up evaluation of PD. Quantitative iron–neuromelanin parameters can enhance the clinical evaluation of Parkinsonism. Additionally, Hartono et al. found that quantitative neuromelanin–iron MRI is associated with PD motor severity and has the potential to enhance diagnostic confidence in clinical settings and monitor PD progression.

In addition to the abnormal alterations in iron deposition and neuromelanin of substantia nigra of PD patients, dynamic functional connectivity has emerged as a valuable biomarker for evaluating the progression of PD (3). Excessive daytime sleepiness (EDS) is a common non-motor symptom in PD patients. Tan et al. discovered that the strong dynamic functional connectivity within and between the SMN and VIS served as an imaging biomarker of EDS in PD patients, potentially reflecting the pathophysiological features of EDS in PD patients.

Brain glucose metabolism was found to be reduced in PD patients with cognitive dysfunction. Sun et al. found decreased glucose metabolism in the frontal and posterior cortex in newly diagnosed and untreated PD patients, indicating that changes in glucose metabolism in specific brain regions can indirectly reflect the level of cognitive function.

Cognitive impairment and autonomic impairment such as orthostatic hypotension (OH) are the most common non-motor symptoms in PD patients (4). Even though the relationship between OH and cognitive dysfunction has been reported, it remains unclear if they are frequently observed simultaneously. Xue et al. used ^18^F-FDG-PET to investigate the relationship between OH and cerebral glucose metabolism in PD patients. Their findings revealed a negative correlation between glucose metabolism in the right medial temporal lobe and delayed recall verbal memory ability. PD patients with OH exhibited poor delayed recall verbal memory function, suggesting that impaired memory function in PD with OH may be attributed to the decreased metabolic function in the medial temporal lobe due to OH.

DAT-PET imaging utilizes ^11^C-methyl-N-2β-carbomethoxy-3β-(4-ffuorophenyl)-tropanel (^11^C-CFT) tracers to evaluate the function of presynaptic dopaminergic neurons in the striatum, providing insight into the severity of dopaminergic neuronal degeneration. This method is valuable for distinguishing PD from essential tremor, dystonia, drug-induced Parkinsonism, and vascular Parkinsonism (5). Fan et al. observed that ^11^C-CFT uptake in the caudate nucleus was higher in the early-onset PD (EOPD) group compared to the late-onset PD (LOPD) group. Furthermore, they found that ^11^C-CFT uptake in the caudate nucleus, as well as the anterior and posterior part of the putamen, was negatively correlated with the age of onset, H&Y stage, disease duration, UPDRS score, UPDRS III score, rigidity score, and bradykinesia score. These findings demonstrate that ^11^C-CFT PET can be used as an important objective biomarker to evaluate disease severity and monitor disease progression.

Total-body PET/CT scanner has the advantage of accurately observing PET tracer biodistribution throughout the entire human body. Xin et al. first utilized the total body PET/CT to study DAT PET biodistribution in a real-time and dynamic mode in major organs, which include kidneys, lungs, spleen, thyroid, heart wall, liver, whole brain, muscle, striatum, and bone. They found that different organs had several unique types of ^11^C-CFT distribution pattern types. ^11^C-CFT was also calculated to be an extremely low radiation dose according to the study result, which is ~2.83E-03 mSv/MBq. It only accounts for one-third of the previous literature that has published radiation dose values, which makes it much more suitable for PD patients to do multiple follow-up examinations.

Multimodality imaging techniques hold significant promise for advancing our comprehension of PD and atypical Parkinsonism. Future research should prioritize investigating the interplay between structural, functional, metabolic, and molecular changes in these conditions to achieve a more comprehensive understanding of the pathological mechanism of PD. Furthermore, larger prospective studies are essential to confirm the diagnostic precision and therapeutic effectiveness of multimodal imaging techniques in PD and atypical Parkinsonism.

## Author contributions

TS: Conceptualization, Supervision, Writing—original draft, Writing—review & editing. BN: Writing—original draft.
